# Nitric oxide and traumatic brain injury

**DOI:** 10.4103/mgr.MEDGASRES-D-25-00357

**Published:** 2026-05-14

**Authors:** Dong Liang, Han Gao, Guoyuan Fan, Tianyu Zhang, Qibing Huang, Hai Yu, Zeli Zhang

**Affiliations:** 1 Department of Neurosurgery, Qilu Hospital of Shandong University, Jinan, Shandong Province, China; 2 Department of Emergency Neurosurgical Intensive Care Unit, Qilu Hospital of Shandong University and Brain Science Research Institute of Shandong University, Jinan, Shandong Province, China

**Keywords:** endothelial nitric oxide synthase, inducible nitric oxide synthase, medical gas, neuroinflammation, neuronal nitric oxide synthase, nitric oxide synthase inhibitor, nitric oxide, secondary brain injury, traumatic brain injury, treatment

## Abstract

Dysregulation of the nitric oxide/nitric oxide synthase system is consistently observed in the aftermath of traumatic brain injury. It plays a pivotal yet paradoxical role in traumatic brain injury progression. Moreover, the modulation of the altered nitric oxide/nitric oxide synthase homeostasis via inhaled nitric oxide, nitric oxide donors, nitric oxide synthase inhibitors, and indirect methods such as mild hypothermia and medical gases in the brain, both during and after traumatic brain injury, can exert a significant impact on the clinical outcome. This review integrates current findings from preclinical studies and clinical trials to elucidate the dynamic contributions of nitric oxide to traumatic brain injury pathophysiology and to highlight emerging interventions targeting nitric oxide-related pathways. While physiological nitric oxide levels support cerebral blood flow and neuroprotection, excessive production via nitric oxide synthase isoforms (especially inducible nitric oxide synthase) drives secondary injury through cytotoxicity, edema, and blood–brain barrier disruption. Therapeutic strategies targeting nitric oxide pathways, including inhaled nitric oxide, nitric oxide donors, and specific nitric oxide synthase inhibitors like VAS203, show significant neuroprotective potential in preclinical models, though clinical translation remains limited.

## Introduction

Traumatic brain injury (TBI) is clinically and pathophysiologically defined as any alteration in brain function or neuropathological state that comes from an external physical force. These forces can range from a mild blow to the head to a very severe blow (for example, in a car accident or a fall from a great height). Globally, an estimated 70 million people are affected yearly.^1^

Primary brain injury in TBI refers to the focal or diffuse neural damage that occurs instantaneously as a direct result of external mechanical forces. These injuries occur concomitantly with the traumatic event. Based on the injury mechanism and anatomical structure, they can be divided into concussion, scalp injury, calvarial fractures, basilar skull fracture, epidural hematoma, subdural hematoma, subarachnoid hemorrhage, intracerebral hematoma, cerebral contusion, diffuse axonal injury, and traumatic cerebrovascular disorders.^2^ In contrast, a secondary brain injury develops after the initial injury and is triggered by delayed cell-mediated events such as ischemia and hypoxia following the primary injury. With this cascade, several other complex pathophysiological changes may occur including cerebral edema, brain herniation, increased intracranial pressure (ICP) and hydrocephalus.^3^,^4^ These detrimental changes may occur through different interrelated pathways, such as disturbance in neurotransmitter systems, ion channel dysfunction, intracellular calcium overload and the release of pro-inflammatory mediators.^5^ Several clinical and physiologic causes can be identified that increase the risk for secondary brain injury (such as failure of cerebrovascular autoregulation, hypoxia, hypercapnia, seizures, fever, cerebral vasospasm, and paroxysmal sympathetic hyperactivity), and cardiac arrhythmias (such as bradycardia, and tachycardia). These risk factors converge on key pathological mechanisms such as altered cerebral blood flow (CBF), brain metabolism, oxidative stress and excitotoxicity, mitochondrial dysfunction or neuroinflammation, leading to neuronal loss and progressive tissue damage.^6^,^7^,^8^ Critically, whereas primary injury is largely irreparable and inevitable, secondary injury can be modifiable.^9^ Consequently, contemporary management of TBI aims at early identification and control of secondary injury; hence, control of secondary injury is central to the neuroprotective treatment options.^10^,^11^,^12^

For many years, nitric oxide (NO) was viewed simply as an environmental pollutant. This assessment changed dramatically with the breakthrough work by Furchgott et al.^13^, whose discovery of NO’s role as a pivotal signaling molecule in vascular smooth muscle relaxation earned them the Nobel Prize in Physiology or Medicine in 1998. Since then, NO has been widely investigated as an important endogenous mediator of almost any physiological function.^14^ Endogenously, NO is produced mainly by three different isoforms of NO synthase (NOS) enzymes: neuronal NOS (nNOS), inducible NOS (iNOS) and endothelial NOS (eNOS). These isoforms catalyze the oxidative conversion of L-arginine to L-citrulline, concomitantly releasing NO as a gaseous product.^14^ The biological activity of NO is greatly dependent on the intracellular calcium ion (Ca^2+^) concentration. The main pathway of NO is the activation of soluble guanylate cyclase, followed by the production of cyclic guanosine monophosphate. Through soluble guanylate cyclase/cyclic guanosine monophosphate, this axis translates a cascade of downstream effects critical for core neurovascular and neuromodulatory functions, including neurotransmitter release, synaptic plasticity and the dynamic regulation of CBF.^15^

This review aimed to conduct a comprehensive and in-depth review, with a specific focus on research pertaining to NO in TBI. It is divided into two associated domains: first, basic research designed to systematically elucidate NO biosynthetic and metabolic pathways in the dynamic pathological microenvironment during TBI; second, clinical investigations that critically evaluate therapeutic interventions targeting NO signaling, as well as translational considerations in this regard. At the same time, this review will cover important roles of NO in the initiation, progression, and neuroremodelling associated with TBI.

## Search Strategy

### Database selection

We systematically searched the PubMed and Web of Science Core Collection databases from inception to February 10, 2026. To minimize the risk of missing eligible studies, we additionally performed manual citation tracking of the reference lists of included articles and relevant reviews to identify further potentially eligible studies. The search was constructed around two core concepts: NO/NOS and TBI. In PubMed, we combined controlled vocabulary terms (e.g., MeSH) with free-text terms in the title and abstract fields, applying Boolean operators (AND/OR), field restrictions, and synonym expansion to optimize both sensitivity and specificity. In Web of Science, we used the Topic field to achieve a balanced trade-off between coverage and precision. The search formula is ((nitric oxide[Title/Abstract]) OR (nitric oxide synthase[Title/Abstract])) AND (traumatic brain injury[Title/Abstract]) AND ((therapy[Title/Abstract]) OR (clinical[Title/Abstract]) OR (mechanism[Title/Abstract])).

Inclusion criteria were original research focusing on mechanistic roles of NO/NOS in the course of TBI; provision of explicit mechanistic evidence; evaluation of NO-related interventions with quantitatively reportable outcomes; and availability of the full text in English. Exclusion criteria were meta-analyses, commentaries, study protocols, letters, and conference abstracts without full text (i.e., non-original research); studies primarily addressing non-traumatic central nervous system (CNS) disorders and irrelevant to the research question; experimental or clinical studies lacking NO-related mechanisms or outcome validation; duplicate publications; and studies with insufficient methodological detail.

### Search and screening process

All records were imported into EndNote for de-duplication. Two investigators independently conducted title/abstract screening and full-text eligibility assessments. Discrepancies were resolved through discussion, with adjudication by a third investigator when necessary. A total of 178 records were identified; 11 were excluded at the title/abstract stage; 9 were excluded at the full-text assessment stage; 14 were manually retrieved, traced and supplemented, and 144 studies were ultimately included for synthesis.

### Data extraction and analysis

Two investigators independently performed a full-text review and extracted data using a standardized form. Extracted items included basic study characteristics; study design and model details; NO-related metrics and detection methods; interventions (type, dose, route/administration or treatment schedule); primary endpoints and measurement methods; key findings; and authors’ conclusions. After cross-verification, data were collated and synthesized using a prespecified thematic framework via narrative integration and comparative analysis.

### Screening and analysis of literature

At the full-text level, we re-verified study relevance and completeness of reporting, excluding articles that were mismatched or methodologically inadequate. For the final included studies, we interpreted the evidence in terms of consistency of findings, reproducibility, and biological plausibility, and distilled coherent evidence chains centered on key mechanisms and outcomes to produce a structured summary and discussion.

## Basic Research on Nitric Oxide in Traumatic Brain Injury

### Role of nitric oxide and nitric oxide synthase in traumatic brain injury

Following TBI, NO has diverse critical physiological effects, which include regulating vascular tone, controlling neurotransmission and mediating anti-inflammatory responses.^16^ Mechanistically, NO, which is a key neurotransmitter,^17^ activates soluble guanylate cyclase, thereby initiating the biosynthesis of cyclic guanosine monophosphate.^18^ Many cell types are able to produce NO, including neurons and vascular endothelial cells. NO plays a key role in several important processes occurring in neuronal cells, such as synaptic plasticity regulation, neural regeneration modulation, N-methyl-D-aspartate receptor (NMDAR) functional tuning and long-term potentiation facilitation.^19^,^20^ In blood vessels, NO is a potent vasodilator: it causes relaxation of vascular smooth muscle cells and drives vascular dilation,^21^ and is crucial for regulating CBF.^22^,^23^,^24^ Under physiological conditions, the cerebral vasculature exhibits a high degree of sophistication and autonomy in pressure regulation. This intrinsic capability is often lost following TBI and causes CBF regulation disturbances. Notably, dysfunction of autoregulation can occur immediately after injury or after a variable latency period, and the duration of such impairment can vary greatly among individuals. Crucially, fluctuations of CBF after TBI are strongly correlated with simultaneous changes in NO concentration. Indeed, NO has an indispensable regulatory role in both the disruption and recovery of CBF after injury.^25^,^26^ It is well known that a decrease in blood pressure exerts a compensatory up-regulation of NO synthesis within the CNS. Such increased NO synthesis later leads to vasodilation, thus ensuring that a sufficient blood supply reaches the brain tissue.^27^,^28^ As a result, basal NO synthesis is crucial to maintain the cerebrovascular tone and to regulate cerebral blood volume. During the acute phase after trauma, endogenous feedback mechanisms are also responsible for further amplifying NO generation to enhance vasodilation and optimize regional CBF. Such an adaptive response not only supports hemodynamic stability but also acts to enhance the functional recovery of reversibly injured neurons in the ischemic penumbra.

Nevertheless, NO also has negative effects in TBI and plays a role in cytoxicity, cerebral oedema and aggravation of neuroinflammatory responses.^29^,^30^ Increasing evidence also suggests that excessive NO can promote various forms of neuronal death in the CNS, and that it can have overtly detrimental effects.^31^ It can even mediate the disruption of the blood–brain barrier (BBB) and contribute to cerebral hyperemia and edema.^32^,^33^,^34^ Another important consequence of TBI is mitochondrial damage. Previous studies have shown that injury-induced mitochondrial damage leads to loss of mitochondrial physiological and metabolic homeostasis, with a subsequent enhancement of cell death.^35^,^36^ One of the main drivers of this cascade is pathological Ca^2+^ influx into the mitochondria, which not only triggers generation of reactive oxygen species, but also induces mitochondrial membrane depolarization, simultaneously reducing adenosine triphosphate generation.^37^,^38^,^39^ As a result, the electron transport chain and oxidative phosphorylation are compromised, which in turn leads to impaired calcium homeostasis and metabolic dysfunction.^40^ NO increase can also affect cytochrome C oxidase, an important terminal enzyme in the mitochondrial respiratory chain, and affect the normal function of mitochondria.^41^ Studies of TBI patients have demonstrated that after TBI, the early concentration of NO in extracellular fluid has a significantly positive relevance to the adverse prognosis and it can be of some value in judging the prognosis of TBI patients.^42^

NO is produced through the oxidation of L-arginine by NOS, and at the same time, citrulline is generated as a byproduct.^43^,^44^ Unlike the classical signaling molecules, NO has no specific storage pool or regulated release mechanism; this limitation is further compounded by the short half-life of NO.^45^ Consequently, the steady-state concentration of NO in target cells is nearly exclusively controlled by its rate of synthesis, which, in turn, is almost absolutely dependent on the rate of NOS enzymatic activity. In most studies examining physiological and pathophysiological roles of NO, NOS activity is measured mainly as a functional proxy.

It is well-established that NOS exists in three distinct isoforms: eNOS, nNOS and iNOS.^46^ All three isoforms are present in the CNS. Among these, eNOS and nNOS are collectively classified as constitutive NOS (cNOS), which are mainly expressed in endothelial cells and neurons, respectively. eNOS is mostly known for regulating vascular dilation and constriction. In contrast, nNOS is involved in the modulation of neuronal physiological functions and the cellular damage following brain injury in the early stage. The expression of iNOS is upregulated by various exogenous or endogenous pro-inflammatory or pathological stimuli, such as damage-associated molecular patterns (DAMPs) and pathogen-associated molecular patterns.^37^ Typically, the cNOS isoforms are relatively inactive under basal conditions and need post-translational modifications for activation. Specifically, binding of extracellular signaling molecules to their cognate cell-surface receptors increases intracellular Ca^2+^, resulting in a rapid increase in intracellular calcium levels. Such elevation also has the effect of allowing cNOS to associate with calmodulin, resulting in immediate enzymatic activation. The classic mechanism of cNOS activation after TBI is the Ca^2+^ influx stimulated by glutamate after neuronal cell membrane depolarization.^38^ By contrast, iNOS is completely independent of both Ca^2+^ and calmodulin and is not post-translational in its activation, resulting in sustained NO production once transcriptionally upregulated.^47^,^48^

NO plays a dual role in TBI. Physiologically, NO regulates vascular tone, neurotransmission, and anti-inflammatory activity, all vital for cerebrovascular homeostasis and to help neuronal recovery after TBI. However, excessive NO production can lead to cytotoxicity, exacerbate brain edema, and disrupt the BBB, contributing to secondary brain injury. The concentration of NO, related to NOS activity, largely determines its beneficial or harmful effects. eNOS and nNOS, as cNOS, are involved in vascular and neuronal regulation, respectively, while iNOS is upregulated in an inflammatory stimulus-dependent manner.^41^ A thorough understanding of the balance between NO’s physiological and pathological roles, along with the precise regulation of NOS isoforms, is critical for developing strategies to attenuate TBI-induced brain damage and improve patient outcomes.

### Role of glutamate in inducing Ca^2+^ responses

Release of DAMPs following mechanical injury of the brain is a key pathophysiological event that regulates NO homeostasis after TBI.^49^,^50^ DAMPs are capable of triggering aseptic inflammatory responses in neurons, which in turn initiate a signal transduction cascade that ultimately results in the activation of cNOS and the upregulation of iNOS. During this complex process, DAMPs trigger the release of pro-inflammatory cytokines, including tumor necrosis factor-α and interleukin-1β.^32^ Soluble tumor necrosis factor-α binds to the tumor necrosis factor receptor 1 (TNFR1) receptor, thereby activating the TNFR1-dependent signaling pathway. This pathway can induce cells to release glutamate into the interstitial fluid and stimulate the extracellular secretion of glutamate.^38^,^51^ Thus, TNFR1 is expressed in almost all the major cell types within the CNS, including microglia, astrocytes and neurons, suggesting that this glutamatergic response may occur in almost all cell types of the neurovascular unit.^51^ Preclinical studies in animals suggested a marked increase in brain tissue glutamate levels after TBI.^52^,^53^ Similarly, in TBI patients, microdialysis was applied to detect the extracellular fluid glutamate concentration in brain tissue. The results showed that the local glutamate concentration significantly increased (up to 1 mM), and the elevated glutamate level was significantly associated with poor prognosis.^54^

Glutamate exerts its multiple physiological and pathological effects via different receptor subtypes, among which is the NMDAR, in particular the NMDA2B subunit (also called NR2B). Increased concentrations of extracellular glutamate activate NMDARs and evoke channel opening, promoting Ca^2+^ influx and intracellular Ca^2+^ overload. This process in turn triggers the activation of neurons expressing nNOS, hence facilitating the synthesis and release of NO.^55^,^56^ The postsynaptic density-95 interacting regulator of spine morphogenesis (Preso), as a special structure on the excitatory postsynaptic membrane of neurons, damages neurons by regulating the NR2B/postsynaptic density-95/nNOS complex.^57^ Downregulation of Preso improves neuronal damage. Although intracellular glutamate can be rapidly cleared by mitochondria through the electron transport chain, extracellular glutamate cannot be cleared this way. Consequently, it will accumulate in the synaptic and perisynaptic spaces and can diffuse to more distal brain regions, where it keeps driving NOS activation. Indeed, accumulating evidence indicates that following TBI, upregulation of various NOS isoforms occurs not only in the ipsilateral injured hemisphere,^27^ but also extends to the contralateral, seemingly unaffected side,^58^ highlighting the widespread and bilateral nature of glutamate-mediated secondary injury mechanisms.

### Role of inducible nitric oxide synthase in neural cells

Under physiological conditions, iNOS remains unexpressed within the CNS. However, during pathological states, a marked upregulation of iNOS occurs in a wide variety of cell types, including neurons, astrocytes, and vascular endothelial cells.^59^,^60^ Once expressed and activated, the activity of iNOS dramatically boosts catalytic capacity for NO production, surpassing cNOS isoforms by several hundred-fold. These effects ultimately lead to a large increase in NO levels that can overwhelm the endogenous antioxidant systems and give rise to cellular dysfunction. These mechanisms include metabolism suppression, induction of cytotoxic cascades, mitochondrial impairment, and ultimately, neuron death in the affected tissue.^61^,^62^

iNOS upregulation after TBI is mainly driven by the sterile inflammatory response triggered by DAMPs released from tissues at the primary injury site. In addition to triggering glutamate release, the aforementioned TNFR1 signaling pathway can also activate the nuclear factor-κB signaling cascade, thereby promoting the upregulation of iNOS. Notably, iNOS is not restricted to a single cell type; robust expression has been documented in astrocytes, microglia, and even neurons under pathological conditions.^63^,^64^,^65^ Within the injured region, two peaks of iNOS expression were observed and each had an associated peak in the local levels of NO. Electron paramagnetic resonance spectroscopy is the most direct method of monitoring NO and has validated the increase in levels of NO in the brain tissue.^66^,^67^ The first burst of iNOS expression after TBI occurs at 4 hours post-injury, and this peak, in contrast to nNOS, is accompanied by a rise in NO levels, as shown by electron paramagnetic resonance measurements.^58^ Temporal correlation between iNOS/NO elevation and immune activation strongly suggests that the immune cells, particularly microglia and macrophages, are the major source of NO during this peak phase in the brain tissue.

Excessive NO reacts chemically with the free radical superoxide to generate peroxynitrite, which is a highly reactive nitrating radical species. This cytotoxic intermediate initiates a cascade of deleterious pathophysiological events, which are rapid cell death, vascular dysfunction and post-translational protein modifications.^68^,^69^,^70^ The formation of peroxynitrite is typically assessed by monitoring the nitration of tyrosine residues within cells and tissues. Studies employing rodent models of TBI have consistently demonstrated a significant upregulation in nitrotyrosine production within cerebral tissue.^71^,^72^ The detrimental effects of iNOS are, in part, attributed to its propensity for easy uncoupling under conditions of low levels of L-arginine and the essential cofactor for NOS, tetrahydrobiopterin. Such an uncoupling phenomenon results in NO and superoxide radicals being easily generated by iNOS.^73^ Unlike nNOS, the characteristic of inducing peroxynitrite generation makes the activation of iNOS more likely to result in an unfavorable prognosis for TBI.

Current evidence shows that iNOS is a double-edged sword,^74^,^75^ and the specific pathological impacts and corresponding outcomes it induces remain a subject of ongoing debate. However, it is clear that iNOS is present and performs its respective role at particular time points and in different regions after TBI.^76^,^77^ Depending on the specific microenvironment and anatomical location within the brain tissue (e.g., the concentrations of peroxides and NO, the levels of antioxidants, the characteristics of target cells, and the stage of disease progression), the reaction between NO and peroxides may give rise to the formation of more noxious substances (such as the aforementioned peroxynitrite). In light of the specificity, delayed expression pattern, and high output rate of NO associated with iNOS, the majority of scholars posit that iNOS is implicated in the delayed injury of nerve cells subsequent to brain trauma and is intricately linked to delayed neuronal death. In some other situations, induction of iNOS and the elevation of local NO levels have antioxidant or iron ion-chelating effects and reduce the level of oxidative stress after TB.^75^,^78^,^79^

### Changes in nitric oxide levels in distant tissues

Aside from disrupting NO homeostasis in the ipsilateral and contralateral sides relative to the injured site by TBI, TBI also regulates NO homeostasis in distal organs, for instance, the heart. Increased ICP after TBI activates the vagus nerve and leads to NO-dependent parasympathetic outflow to the heart and other extracranial tissues.^80^ Clinically, this manifests during the acute phase as profound hypotension, a parasympathetic-driven response that has been consistently linked to increased mortality and poor neurological outcomes.^81^ In addition to remote modulation of NOS activity at both transcriptional and post-translational levels, NO generated within the injured brain parenchyma can disseminate systemically via two primary routes: passive diffusion through neural tissue and entry into the systemic circulation. The diffusion rate of NO in brain tissue is remarkably rapid,^82^,^83^ with a maximal diffusion radius extending up to 200 μm.^84^ After TBI, disruption of the BBB allows the release of NO into the circulation. Once in circulation, NO is quickly stabilized by binding to hemoglobin to form two predominant adducts: S-nitrosohemoglobin and iron-nitrosyl hemoglobin. These nitrosohemoglobin complexes are also bioactive reservoirs that can deliver NO to peripheral tissues. In the cardiovascular system, they modulate key physiological processes, including myocardial contractility and relaxation, as well as vascular tone, thereby linking central neuropathology to systemic autonomic and hemodynamic dysfunction after TBI.^85^,^86^

In conclusion, NO has dual functions in TBI in basic research. Physiologically, it maintains vascular tone, neurotransmission, and anti-inflammatory activity to assist neuronal recovery. Pathologically, too much NO produces cytotoxicity, brain swelling and BBB injury. NO’s concentration, tied to NOS activity, determines its effects. Glutamate release after TBI activates NMDARs, leading to Ca^2+^ influx and nNOS activation, promoting NO synthesis. iNOS is up-regulated in pathology and produces NO excessively; excess NO serves to suppress cellular suppression and cytotoxic responses. iNOS excess NO can form peroxynitrite and cause cell death and impairment of the vasculature. TBI also influences the NO homeostasis in distant organs like the heart via vagus nerve stimulation and NO diffusion or release into the bloodstream, affecting myocardial function and vasodilation.

## Role of Nitric Oxide-Related Therapy in the Treatment of Traumatic Brain Injury

NO and its bioactive derivatives play a pivotal role in the pathogenesis and progression of TBI and their expressions are strongly associated with clinical performance and long-term prognosis. Both clinical and fundamental research have demonstrated that some of the treatments used in the clinical treatment of TBI provide a neuroprotective effect through the modulation of NO and NOS pathways. A wide number of studies have dedicated efforts to improving the neurological functional outcomes after TBI by intervening directly into the NO or the NOS signaling pathways (**Tables 1** and **2**). On the other hand, it should also be noted that most of these intervention strategies still exist only in the laboratory stage and have not yet been successfully implemented on a large scale in clinical practice.

**Table 1:** A summary of NO-related therapy in the treatment of TBI

Study	Year	Object	Results
**Inhaled nitric oxide**			
Terpolilli et al.^87^	2013	Mice	Inhaled NO provides neuroprotection following TBI by selectively dilating resistance vessels, which enhances CBF in the traumatic penumbra, ultimately reducing brain damage and improving neurological function
Liu et al.^88^	2013	Mice	Inhaled NO administered post-mTBI accelerates recovery of short-term memory and suppresses neuroinflammation by inhibiting the activation of astrocytes and microglia
Pastor et al.^89^	2019	Pigs	Inhaled NO preserves K^+^ channel-mediated pial artery vasodilation by blocking the sequential upregulation of ET-1, ERK, MAPK, and IL-6, thereby preventing impaired cerebral autoregulation and subsequent hippocampal histopathology after TBI
Price et al.^90^	2024	Humans and Pigs	In TBI patients and porcine model, inhaled NO exerts no significant effects on clinically relevant pulmonary parameters or intracranial dynamics, including ICP, CPP, and CBF. The lack of effect was consistent across both clinical and preclinical settings
Papadimos et al.^91^	2009	Humans	In patients with concurrent TBI and ARDS, administration of inhaled NO was associated with improved clinical outcomes
Vavilala et al.^92^	2001	Humans	In a TBI patient, inhaled NO demonstrated cerebrovascular safety by causing no clinically significant changes in ICP, middle cerebral artery velocity, or jugular venous oxygen saturation
**Nitric oxide donor**			
Lu et al.^94^	2003	Rats	The NO donor DETA/NONOate promotes neurogenesis by significantly enhancing the proliferation, survival, migration, and differentiation of neural progenitor cells in key brain regions, which is associated with improved neurological functional recovery post-TBI
Zhang et al.^95^	2002	Rats	The NO donor S-nitroso-N-acetylpenicillamine reverses the TBI-induced attenuation of hypercapnic vasodilation, whereas the NO-independent vasodilator papaverine is ineffective, suggesting a specific deficit in the NO pathway
Liu et al.^97^	2024	Mice	The injectable MINOR scaffold provided sustained NO release and ROS scavenging, thereby promoting angiogenesis and neuroregeneration, suppressing inflammation, and enhancing post-TBI recovery
**Nitric oxide synthase inhibitor**			
Zheng et al.^115^	2024	Mice	Propofol alleviated TBI-induced ferroptotic injury, reduced neuroinflammation and neuronal damage, and improved long-term neurological outcomes via activation of the eNOS/NO signaling pathway
Schwarzmaier et al.^100^	2015	Mice	Excess NO production from eNOS is the primary driver of the pathological arteriolar vasodilation observed after TBI, a mechanism that is effectively blocked by the NOS inhibitor VAS203
Terpolilli et al.^99^	2009	Mice	The novel NOS inhibitor VAS203 prevents the formation of post-traumatic brain edema and the subsequent rise in ICP, leading to sustained functional improvements
Wang and Han^108^	2018	Mice	The selective iNOS inhibitor 1400W rescues TBI-induced impairment of long-term potentiation in the hippocampus and prefrontal cortex, which underlies the observed improvements in cognitive and motor functions
Mésenge et al.^112^	1996	Mice	The NOS inhibitors L-NAME and 7-NI attenuate post-traumatic neurological deficits, an effect that is reversed by the NO precursor L-arginine, confirming the involvement of NO in the early injury cascade
Tegtmeier et al.^102^	2020	Humans	This phase III study was designed to evaluate whether a 48-h intravenous infusion of Ronopterin improves 6-mon neurological outcome and intracranial physiological parameters in patients with moderate-to-severe TBI
Stover et al.^101^	2014	Humans and Pigs	In a Phase IIa trial with TBI patients, the NOS inhibitor VAS203 significantly improved the 6-mon functional outcome and reduced the required therapy intensity level, despite having no significant direct effect on ICP or CPP
Louin et al.^109^	2006	Rats	Inhibition of iNOS provides a significant neuroprotective benefit by reducing neurological deficits, a therapeutic effect that does not rely on the reduction of cerebral edema
Lu and Moochhala^104^	2004	Rats	Aminoguanidine reduces TBI-induced lesion volume, attenuates neuronal apoptosis, and improves neurobehavioral deficits
Hortobágyi et al.^113^	2003	Rats	Inhibition of nNOS prevents the downstream activation of PARP, while direct PARP inhibition reduces lesion volume, confirming the neurotoxic role of the nNOS-PARP signaling cascade in TBI
Wada et al.^103^	1998	Rats	Inhibition of iNOS with aminoguanidine significantly reduces cortical neuronal necrosis following TBI
Wada et al.^114^	1998	Rats	Selective inhibition of nNOS before injury is neuroprotective, whereas non-selective NOS inhibition L-NAME, is ineffective. Conversely, post-injury administration of L-arginine is also protective, highlighting the differential roles of NOS isoforms in TBI
**Nitric oxide synthase immune intervention**			
Sharma et al.^117^	2006	Rats	Intracerebral administration of a nNOS antiserum within 1 h post-TBI significantly attenuates brain edema, BBB permeability, and sensory-motor deficits
Xu et al.^116^	2018	Rats	Suppression of cortical TRPM7 provides neuroprotection by activating the Akt/eNOS pathway, which in turn attenuates oxidative stress, apoptosis, and brain damage, leading to improved functional outcomes
**Nitric oxide synthase associated neuroinflammation intervention**			
Ismael and Ishrat^119^	2022	Mice	Post-injury C21 treatment improved neurological function and attenuated early inflammatory and apoptotic responses after TBI, accompanied by increased IL-10 expression and eNOS phosphorylation
**Indirect nitric oxide/nitric oxide synthase intervention measures**			
Park et al.^142^	2026	Rats	Mirodenafil reduced neuronal loss after TBI by maintaining the NO–cGMP pathway, suppressing zinc accumulation, and alleviating oxidative stress–related injury
Keshavarzi et al.^126^	2021	Rats	Auraptene at 25 mg/kg attenuated diffuse TBI-induced injury, reducing brain edema, oxidative stress markers, NO, and TNF-α while improving neurological scores
Liang et al.^139^	2021	Rats	HBOT promotes a shift in microglial polarization from the pro-inflammatory M1 phenotype toward the anti-inflammatory M2 phenotype, characterized by suppressed iNOS expression and enhanced Arg1 levels
Wei et al.^143^	2014	Rats	Ebselen confers dose-dependent neuroprotection by concurrently reducing NO levels and inhibiting the TLR4-mediated p38 MAPK signaling pathway, leading to the amelioration of brain edema and neurological deficits
Cope et al.^141^	2012	Rats	Zinc supplementation administered after TBI improves cognitive function, and when combined with an early injection, alleviates depression-like behaviors
Cope et al.^140^	2011	Rats	Pre-injury dietary zinc supplementation significantly improves cognitive performance and attenuates depression-like behaviors and physiological stress responses following TBI
Kuo et al.^131^	2011	Rats	Retrograde cold saline infusion significantly reduces cerebral contusion volume and ameliorates motor deficits, a therapeutic effect attributed to its potent suppression of both post-traumatic oxidative and nitrostative brain damage
Chatzipanteli et al.^129^	1999	Rats	The neuroprotective mechanism of hypothermia involves a multi-faceted modulation of NOS, including the suppression of acute cNOS hyperactivation, preservation of chronic cNOS activity, and inhibition of delayed iNOS induction
Sakamoto et al.^130^	1997	Rats	Mild hypothermia completely abolishes the acute surge in NO synthesis observed immediately following TBI
Liu et al.^144^	2025	Mice	Neuron-targeted GSNOR overexpression reduced GSNO/NO accumulation and ERO1α S-nitrosylation, attenuated endoplasmic reticulum stress and neuronal death, and alleviated secondary brain injury after experimental TBI
Shirasath et al.^124^	2025	Mice	Plumbagin alleviates mTBI-induced obsessive-compulsive-like behaviors by inhibiting nNOS, which reverses neurodegenerative changes and augments cortico-striatal serotonin levels
Chaturvedi et al.^135^	2025	Humans	Early adjuvant HBOT significantly improved discharge GCS and 3-month GOS-E in adults with moderate TBI compared with standard care alone
Ren et al.^137^	2023	Humans	Adjunctive HBOT increased serum SOD, nitric oxide, and NGF levels while reducing MDA, indicating attenuation of systemic oxidative stress after craniocerebral injury
Zhou et al.^138^	2025	Neurons and astrocytes	HBOT attenuates TBI-induced neurotoxicity by inhibiting the expression of TNFR1, leading to the subsequent downregulation of both nNOS and iNOS, and a reduction in NO levels

1400W: N-[3-(aminomethyl)benzyl]acetamidine (a selective inducible nitric oxide synthase inhibitor); 7-NI: 7-nitroindazole; Akt: protein kinase B; ARDS: acute respiratory distress syndrome; Arg1: arginase-1; BBB: blood–brain barrier; C21: compound 21 (an angiotensin II AT2 receptor agonist); CBF: cerebral blood flow; cGMP: cyclic guanosine monophosphate; cNOS: constitutive nitric oxide synthase; CPP: cerebral perfusion pressure; DETA/NONOate: (Z)-1-[N-(2-aminoethyl)-N-(2-ammonioethyl)amino]diazen-1-ium-1,2-diolate (an nitric oxide donor); DTX1: deltex E3 ubiquitin ligase 1; eNOS: endothelial nitric oxide synthase; ER: endoplasmic reticulum; ERK: extracellular signal-regulated kinase; ERO1α: endoplasmic reticulum oxidoreductase 1 alpha; ET-1: endothelin-1; GCS: Glasgow Coma Scale; GOS-E: Glasgow Outcome Scale-Extended; GSNO: S-nitrosoglutathione; GSNOR: S-nitrosoglutathione reductase; HBOT: hyperbaric oxygen therapy; ICP: intracranial pressure; IL-10: interleukin-10; IL-6: interleukin-6; iNOS: inducible nitric oxide synthase; L-NAME: N&apos;-nitro-L-arginine methyl ester hydrochloride; MAPK: mitogen-activated protein kinase; MDA: malondialdehyde; MINOR: mussel-inspired nitric oxide-release microreservoir; mTBI: mild traumatic brain injury; NGF: nerve growth factor; nNOS: neuronal nitric oxide synthase; NO: nitric oxide; NOS: nitric oxide synthase; PARP: poly ADP-ribose polymerase; ROS: reactive oxygen species; SOD: superoxide dismutase; TBI: traumatic brain injury; TLR4: Toll-like receptor 4; TNF-α: tumor necrosis factor-α; TNFR1: tumor necrosis factor receptor 1; TRPM7: transient receptor potential melastatin 7; VAS203: 4-amino-tetrahydrobiopterin (a nitric oxide synthase inhibitor).

**Table 2:** Classification of NO-related therapeutic interventions for traumatic brain injury

Intervention category	Intervening measure
NO	Inhaled NO, NO donor (DETA/NONOate, SNAP, pGSNO, MINOR)
NOS inhibitor	VAS203, AG, 1400W, L-NIL, L-NAME, 7-NI
NOS immune intervention	iNOS antisense oligonucleotides, shRNA specifically targeting TRPM7, nNOS antiserum
NO/NOS and neuroinflammation inhibitor	DTX1
Indirect NO/NOS intervention measures	Plumbagin, CGRP, auraptene, mild hypothermia, H_2_S, HBOT, zinc ions, mirodenafil, ebselen, GSNOR

1400W: N-(3-(aminomethyl)benzyl)acetamide; 7-NI: 7-nitroindazole; AG: aminoguanidine; CGRP: calcitonin gene-related peptide; DETA/NONOate: (Z)-1-[N-(2-aminoethyl)-N-(2-ammonioethyl)amino]diazen-1-ium-1,2-diolate; DTX1: deltex E3 ubiquitin ligase 1; GSNOR: S-nitrosoglutathion reductase; H_2_S: hydrogen sulfide; HBOT: hyperbaric oxygen therapy; iNOS: inducible nitric oxide synthase; L-NAME: NG-nitro-L-arginine methyl ester; L-NIL: L-N-(1-iminoethyl)-l-lysine; MINOR: mussel-inspired nitric oxide-release microreservoir; NO: nitric oxide; nNOS: neuronal nitric oxide synthase; NOS: nitric oxide synthase; pGSNO: poly(S-nitrosoglutathione); shRNA: short hairpin RNA; SNAP: S-nitroso-N-acetylpenicillamine; TRPM7: transient receptor potential melastatin 7; VAS203: 4-amino-tetrahydrobiopterin.

### Inhaled nitric oxide

Following the U.S. Food and Drug Administration’s approval of inhaled NO (iNO) in 1999 for the treatment of persistent pulmonary hypertension of the newborn, iNO has demonstrated its therapeutic efficacy across a spectrum of diseases. Notably, emerging evidence suggests that iNO may also confer neuroprotective benefits in TBI. In a murine model of TBI, Terpolilli et al.^87^ reported that iNO treatment significantly improved CBF and reduced ICP. 24-hour iNO could reduce the volume of the injury lesion, alleviate brain edema and BBB damage, and improve the prognosis of neurological function, demonstrating neuroprotective effects. Subsequent work in a mouse model of mild TBI extended these findings, demonstrating that iNO could ameliorate neuroinflammatory responses and mitigate memory deficits.^88^ The translational relevance of these observations has been explored in larger animal models. Using a porcine TBI model, Pastor et al.^89^ found that iNO preserved potassium channel function and restored impaired cerebral vascular autoregulation, thereby limiting secondary brain injury. However, not all studies have yielded positive results. Price et al.^90^ explored the effects of iNO in both human and porcine TBI models but failed to observe any beneficial outcomes. Their findings demonstrated that iNO did not improve lung function indicators and had no significant impact on ICP and cerebral perfusion pressure. In TBI patients with concomitant acute respiratory distress syndrome or pulmonary hypertension, two case reports documented marked improvements in pulmonary parameters, including reduced pulmonary arterial pressure and enhanced oxygenation, without adverse effects on ICP.^91^,^92^

In summary, iNO has significant therapeutic effects in the management of TBI. iNO specifically improves CBF in TBI patients and alleviates brain injury, with very strong neuroprotective effects. Notably, these benefits are achieved without significantly causing a change in ICP or cerebral perfusion pressure. And as can be seen from the recent studies, for TBI patients complicated by pulmonary hypertension or acute respiratory distress syndrome, iNO can extremely improve pulmonary functions.^93^

### Nitric oxide donor

Lu et al.^94^ carried out an investigative study utilizing a rat model of TBI. In this study, after TBI, exogenous NO donor (Z)-1-[N-(2-aminoethyl)-N-(2-ammonioethyl)amino]diazen-1-ium-1,2-diolate was injected intraperitoneally daily. Their findings revealed that compared to the control group, the experimental cohort exhibited significantly enhanced proliferation, migration, and differentiation of neural precursor cells in key regions such as the hippocampus, striatum, and corpus callosum. Furthermore, these animals demonstrated markedly improved neurological outcomes, suggesting that this NO donor exerts robust neuroprotective effects following TBI. Zhang et al.’s study^95^ on a rat model of TBI showed that the reactivity of cerebral blood vessels to CO_2_ was significantly reduced after TBI. S-nitroso-N-acetylpenicillamine is an NO donor. Local perfusion of S-nitroso-N-acetylpenicillamine can restore the reactivity of cerebral blood vessels to hypercapnia, which has potential application value in the clinical treatment of TBI. Additionally, Chiang et al.^96^ validated the synergistic benefits of combining NO delivery with electrical stimulation *in vivo*. This dual strategy suppressed inflammation, promoted angiogenesis, and mitigated neuronal damage following TBI, highlighting its therapeutic promise. Further advancements have been made through the development of a mussel-inspired NO-release microreservoir, which embeds an NO donor to facilitate sustained NO release. This method has shown efficacy in promoting angiogenesis and reducing inflammatory responses in a mouse TBI model, thereby offering a novel avenue for targeted therapy.^97^ Although these promising preclinical findings do exist, one should stress that the use of these NO donors is so far limited to basic research. None of these interventions so far has successfully translated into clinical practice; there is a need for solid translational studies for their safety and efficacy in humans.

### Nitric oxide synthase inhibitor

At present, a considerable number of animal TBI model experiments have been conducted to study the effects of NOS inhibitors.^98^ The NOS inhibitors that have been more extensively studied are VAS203, aminoguanidine dihydrochloride (1400W), L-N-imidoethyllysine (L-NIL), N’-nitro-L-arginine methyl ester hydrochloride (L-NAME) and 7-nitroindazole (7-NI).

#### VAS203

VAS203, also known as Ronopterin, is a novel NOS inhibitor that can rapidly reduce the NO levels in cerebral vessels. This particular mechanism holds substantial therapeutic promise for the management of conditions associated with an overproduction of NO. Terpolilli et al.^99^ successfully established a rat model of TBI and subsequently administered VAS203, intravenously to the experimental cohort. The experimental findings revealed that, in comparison to the control group, VAS203 administration effectively mitigated brain tissue edema, attenuated the elevation of ICP, and ameliorated the deterioration of CBF. Furthermore, it significantly enhanced neurological functional recovery. These results collectively suggest that VAS203 holds promise as a potential therapeutic intervention for managing acute ICP elevation following TBI. Schwarzmaie et al.^100^ studied the mouse TBI model and administered VAS203 intravenously to the experimental group. The results showed that increased eNOS levels were the cause of small artery dilation, which could lead to increased cerebral blood volume and ICP, resulting in a poor prognosis. Notably, the experimental group that received intravenous VAS203 demonstrated the capacity to mitigate these detrimental effects by inhibiting the dilation of small cerebral arteries. Consequently, VAS203 emerges as a promising therapeutic candidate for the management of TBI. The NOSTRA Phase II clinical trial investigated the effect of VAS203 in TBI patients. This study collected 32 TBI patients from six centers in Europe and administered low, medium and high doses of VAS203 intravenously to the experimental group. The results showed that there were no significant differences in ICP, cerebral perfusion pressure and cerebral metabolism between the experimental group and the placebo group. However, the treatment intensity of the experimental group was significantly lower than that of the placebo group 6 days after injury, and the extended Glasgow Outcome Score of the experimental group was significantly higher than that of the placebo group at 6 months, suggesting that VAS203 has a neuroprotective effect after TBI and can improve the clinical prognosis of patients. However, it should be noted that high-dose application of VAS203 carries the risk of acute renal function impairment.^101^ Building on these results, the ongoing NOSTRA Phase III trial, a randomized, double-blind, placebo-controlled, multicenter study, is currently assessing the efficacy and safety of VAS203 in a larger cohort of moderate-to-severe TBI patients. Although results remain pending,^102^ VAS203 stands as one of the most advanced NOS-targeted therapeutics in TBI. While still investigational, it holds considerable promise as a future cornerstone in multimodal neurocritical care, if confirmed effective and safe in definitive clinical trials.

#### 1400W and L-NIL

Aminoguanidine, a well-characterized selective inhibitor of NOS, primarily exerts its effect by diminishing NO production through the suppression of NOS activity. Notably, its inhibitory action is particularly pronounced on iNOS. Wada et al.^103^ conducted a comprehensive study utilizing a rat model of TBI, revealing that the expression of iNOS exhibited a time-dependent pattern following TBI. The intraperitoneal administration of aminoguanidine effectively curbed the expression of iNOS and markedly reduced the quantity of necrotic neurons in the cerebral cortex, thereby demonstrating its neuroprotective properties. These findings were further corroborated by Lu and Moochhala.^104^ Using a similar rat TBI model, they reported that aminoguanidine could mitigate brain damage subsequent to TBI, decrease the number of apoptotic neurons, and enhance the prognosis of neurological function.

1400W is characterized as a slow-acting, tightly binding, and highly selective inhibitor of iNOS.^105^ Conversely, L-NIL serves as an efficacious, moderately selective iNOS inhibitor that specifically targets and inhibits iNOS activity, thereby attenuating excessive NO production. The primary mechanism of action for L-NIL involves its binding to the active site of iNOS, which obstructs the entry of the substrate L-arginine and consequently inhibits NO synthesis.^106^,^107^ In a murine model of TBI, Wang and Han^108^ demonstrated that the immediate intravenous administration of 1400W following TBI could mitigate the prominent remodeling damage associated with TBI. This intervention was accompanied by measurable improvements in both motor coordination and cognitive performance, highlighting its neuroprotective efficacy. Further comparative insights were provided by Louin et al.,^109^ who employed a rat TBI model to evaluate the therapeutic effects of three distinct NOS inhibitors (aminoguanidine, L-NIL, and 1400W) on BBB integrity, cerebral edema, and neurological function. Their results revealed that while aminoguanidine notably reduced brain edema, all three compounds conferred significant improvements in neurological outcomes. These findings collectively underscore the therapeutic relevance of selectively targeting iNOS in mitigating secondary injury cascades following TBI.

#### L-NAME and 7-NI

L-NAME is a widely used non-selective inhibitor of NOS that suppresses NO production by broadly inhibiting NOS enzymatic activity. In contrast, 7-NI is a potent, competitive, and relatively selective inhibitor with preferential activity toward nNOS.^110^,^111^ Mésenge et al.^112^ investigated the impact of L-NAME and 7-NI on neurological dysfunction in a murine model of moderate TBI. Their findings demonstrated that when administered at 5, 30, or 60 minutes post-injury, both compounds significantly ameliorated neurological deficits assessed at 24 hours, indicative of acute neuroprotective efficacy. However, this benefit was lost when treatment was delayed to 2 hours post-TBI, suggesting a narrow therapeutic window for NOS inhibition in the early phase of secondary injury. These observations are further supported by independent preclinical studies,^99^,^100^ who reported that post-TBI administration of either L-NAME or 7-NI reduced lesion volume and attenuated histopathological damage, reinforcing their potential as neuroprotective agents.^113^,^114^ Notably, recent evidence has also linked NOS modulation to other cytoprotective mechanisms. For instance, propofol, a commonly used intravenous anesthetic, has been shown to mitigate ferroptosis and improve outcomes after TBI through activation of the eNOS/NO signaling pathway. Crucially, this neuroprotective effect of propofol was abolished upon co-administration of L-NAME, confirming the functional dependence of propofol’s action on intact NO signaling.^115^ Together, these findings illustrate the complex and context-dependent roles of the NOS isoforms in TBI pathophysiology and indicate the importance of timing, selectivity and downstream signaling when therapeutic targeting is aimed at.

So far, none of the aforementioned pharmacotherapeutic agents have been integrated into routine guidelines for the management of TBI. Nevertheless, a strong preclinical evidence base, from a wide range of animal models, reliably points to their neuroprotection potential, indicating their capacity to abrogate neuronal damage and promote functional recovery following TBI. Of these candidates, the most clinically advanced is VAS203, which has advanced to clinical trials in humans, a milestone bridging mechanistic promise to clinical feasibility. Currently ongoing Phase III NOSTRA trial also makes visible the translational excitement for this compound. With the very encouraging preclinical evidence and the ongoing clinical trial of VAS203, there is ample well-founded optimism that targeted modulation of NO signaling will eventually lead to effective, mechanistically rational therapies for TBI. Considerable hurdles still exist in translating bench findings to bedside application, but these agents point toward a promising territory in the quest for disease-modifying drugs in neurotrauma.

### Nitric oxide synthase immune intervention

The primary injury resulting from TBI induces innate immune activation and modifications in cerebral metabolism, thereby triggering secondary pathological cascades that may exacerbate secondary injury. Immunological interventions targeting NO and NOS following TBI can also confer neuroprotective effects by suppressing NO levels. In a rat model of TBI, Huttemann et al.^41^ demonstrated that intraventricular delivery of iNOS-targeted antisense oligonucleotides effectively suppressed iNOS mRNA expression, restored cytochrome C oxidase activity, and fully normalized adenosine triphosphate levels, providing compelling evidence for the neuroprotective potential of selective iNOS inhibition. Complementing this approach, Xu et al.^116^ reported that cortical administration of viral vectors encoding short hairpin RNA against transient receptor potential channel 7 attenuated brain edema, reduced contusion volume, and improved neurological function. These benefits were mechanistically linked to activation of the Akt/eNOS signaling axis, suggesting that strategic modulation of eNOS activity, rather than blanket suppression, may also be therapeutically advantageous. Further underscoring the importance of isoform- and time-specific targeting, Sharma et al.^117^ investigated the effects of intracerebral nNOS antiserum in a rat TBI model. When administered at 5 minutes or 1 hour post-injury, the antiserum significantly reduced nNOS expression, alleviated cerebral edema, and enhanced sensorimotor recovery. However, no appreciable benefit was observed with delayed administration at 2 hours, highlighting a narrow therapeutic window for nNOS-directed interventions. Collectively, these studies illustrate that immunomodulatory strategies targeting specific NOS isoforms can confer significant neuroprotection, but their efficacy is critically dependent on both molecular selectivity and precise timing. This reinforces the need for rigorously defined intervention windows in the design of future clinical trials aimed at harnessing NO pathway modulation for TBI therapy.

### Nitric oxide synthase-associated neuroinflammation intervention

The inflammatory response triggered by TBI represents a sterile immune reaction, initiated primarily through purinergic signaling pathways activated by extracellular adenosine triphosphate released from damaged or dying cells. This cascade recruits and activates key innate immune effectors, including microglia, infiltrating monocytes, and neutrophils, thereby orchestrating the early neuroinflammatory milieu.^49^,^118^,^119^ As the resident immune cells within the CNS, microglia exhibit a rapid and immediate response to inflammatory stimuli.^120^,^121^ Upon sensing purinergic signals emanating from compromised tissue, they swiftly transition into an active phagocytic state, facilitating debris clearance and limiting secondary damage, functions that are integral to their neuroprotective capacity. Critically, microglial functional polarization exerts a profound influence on TBI pathophysiology. For instance, Zhang et al.^122^ demonstrated that overexpression of Deltex E3 ubiquitin ligase 1 promoted proinflammatory cytokine production and shifted microglia toward the M1 phenotype, as evidenced by elevated iNOS and reduced arginase-1 in TBI model rats.

### Indirect nitric oxide/nitric oxide synthase intervention measures

NO/NOS not only regulates the secondary injury of TBI through iNO and NO donor and other direct methods, but also indirectly regulates the development of TBI and its prognosis. As such, this pathway is intricately involved in the pathophysiology of TBI and provides a treasured field for a number of treatment opportunities.

#### Nitric oxide/nitric oxide synthase as regulating factors for traumatic brain injury

As research into TBI advances, a growing body of evidence implicates NO and NOS, not merely as a bystander but as a regulator, in some plant extracts mediated pathophysiological cascade following TBI.^123^ Specifically, Shirasath et al.^124^ proved that plumbagin alleviates obsessive-compulsive disorder-like behaviors induced by mild TBI in mice, and the concentrations of NO were significantly decreased in the medial prefrontal cortex and striatum, with the underlying mechanism involving the inhibition of nNOS. Additionally, Calcitonin gene-related peptide demonstrates neuroprotective effects in acute brain injuries. The expression of nuclear factor erythroid-2-related factor 2 and eNOS was decreased in both male and female rat TBI models, treated with Calcitonin gene-related peptide inhibition.^125^ Moreover, TBI-induced brain damage was significantly inhibited by Auraptene, decreased NO levels and oxidative stress inhibition were detected in Auraptene treated rat TBI model.^126^

#### Mild hypothermia

Therapeutic mild hypothermia (33–35°C) is well recognized for its neuroprotective role in TBI and for its reduction of ICP, its decrease in mortality in severe brain injury and for its beneficial long-term neurological effect.^127^,^128^ Our multifaceted intervention operates via a network of interconnected mechanisms, many of which include modulation of NOS isoforms as one of the main features that contribute to its effectiveness in combating secondary brain injury.

In a rat model of TBI, Chatzipanteli et al.^129^ demonstrated that moderate hypothermia attenuates early cNOS activity while simultaneously blunting the delayed upregulation of iNOS, a dual effect that helps constrain excessive NO production during critical post-injury phases. Complementing these findings, Sakamoto et al.’s investigation^130^ revealed that mild hypothermia could diminish the extracellular concentrations of nitrate and nitrite in the locally damaged region of TBI. Additional mechanistic insights were provided by Kuo et al.,^131^ who established a TBI model and induced brain hypothermia by infusing 4°C saline through the jugular vein. The findings in the above study demonstrated that hypothermia could markedly reduce the volume of contused brain tissue, lower nNOS levels, alleviate oxidative stress, and enhance the prognosis of neurological function. Spreading depolarization represents a well-documented neurophysiological phenomenon, marked by the propagation of waves of neuronal depolarization across brain tissue. These waves induce transient disruptions in ionic gradients, subsequently resulting in a temporary suppression of neuronal activity. Notably, the hyperemia that is typically induced by spreading depolarization can be effectively mitigated through focal brain cooling techniques. Furthermore, empirical evidence suggests that the suppression of spreading depolarization is intricately linked to the downregulation of endothelial eNOS expression in rat models.^132^ Taken together, these observations underscore that mild hypothermia exerts neuroprotective effects by reducing both NOS content and NO levels in the localized area of TBI-induced damage.

#### Other medical gases

Given their facile delivery, rapid absorption into systemic circulation, the ease of traversing the BBB, and subsequent distribution throughout brain tissue, inhalational gases, such as hydrogen sulfide and hyperbaric oxygen, with neuroprotective properties, represent an invaluable therapeutic resource for the clinical management of patients with TBI. Accumulating evidence has elucidated that these gaseous molecules confer neuroprotection via interactions with the NO/NOS signaling pathways.

*Hydrogen sulfide*

Hydrogen sulfide plays an important role in neurotransmission, regulation of vascular tone, angiogenesis, inflammation, and apoptosis and exhibits a pronounced neuroprotective effect in TBI.^133^ It has been demonstrated that hydrogen sulfide-releasing peptides (SVRN-4) reduced NO-induced tissue damage in a mouse model of weight loss TBI.^134^ However, for this particular research, no additional functional studies were carried out through animal experiments or clinical trials.

*Hyperbaric oxygen*

The therapeutic rationale for hyperbaric oxygen therapy in TBI lies in its capacity to attenuate the cascade of secondary brain injury triggered by the initial primary mechanical trauma.^135^,^136^,^137^ Zhou et al.^138^ presented that TNFR1 knockdown and hyperbaric oxygen enhanced neuronal viability, decreased astrocyte viability in an *in vitro* model of TBI, in which levels of NO, nNOS and iNOS were tested down-regulated. TBI elicited a marked and time-dependent upregulation in the expression levels of both the M1 phenotype marker, iNOS, and the M2 phenotype marker, arginase-1. Notably, hyperbaric oxygen therapy exerted a differential regulatory effect, significantly attenuating the TBI-induced elevation in iNOS expression, while concurrently augmenting the expression level of arginase-1.^139^

#### Zinc ions

Physiological concentrations of zinc ions, along with zinc-containing compounds like zinc chloride and zinc protoporphyrin, exhibit neuroprotective properties on neurons. This neuroprotective effect is, in part, associated with the influence of zinc ions on the levels of neuronal nNOS in the hippocampus. Zinc ions can bind to the subunits of NMDARs. Following TBI, this binding reduces the excessive influx of Ca^2+^ that is induced by glutamate and mediated by NMDARs. As a consequence, the intracellular Ca^2+^ concentration decreases. Since the activity of Ca^2+^-dependent NOS is inhibited by the reduced intracellular Ca^2+^ levels, there is a subsequent decrease in NO production. This reduction in NO production ultimately alleviates the neurotoxic effects that are induced by excessive NO.^140^,^141^ In addition, Park et al.^142^ demonstrated that mirodenafil has protective effects on TBI-induced neuronal death in rat TBI models and the possible mechanism is to promote the accumulation of zinc and synthesis of NO.

#### Ebselen

Wei et al.^143^ conducted an experiment wherein Ebselen was administered to a rat model of TBI through gastric intubation. The findings revealed that treatment with Ebselen markedly ameliorated neuroinjury in TBI rats, with the extent of improvement exhibiting a dose-dependent relationship. It significantly reduced the levels of NO and iNOS mRNA and inhibited the expression of Toll-like receptor 4 and phosphorylated p38 mitogen-activated protein kinase, indicating that the NO and p38 mitogen-activated protein kinase signaling pathways are involved in the neuroprotective effect of ebselen.

#### Others

S-Nitrosoglutathion reductase overexpression adeno-associated virus was constructed and administered to mice by stereotactic cortical injection, decreased S-nitrosoglutathion and NO and improved short-term neurobehavioral outcomes in mice.^144^

Current work highlights the important role played by NO and NO-derived compounds in TBI pathophysiology and prognosis and various therapeutic targets of NO and NOS to treat it. Nevertheless, most of these therapies are only applied in basic research and have not been promoted in large trials for clinical translation. Key areas are iNO, which can increase CBF and decrease brain damage without provoking significant effects on ICP, but results are inconsistent between the available studies. NO donors have the ability to increase neurogenesis and also induce vascular reactivity. The NOS inhibitors VAS203, 1400W and L-NAME exhibit neuroprotection in animal models and, in particular, VAS203 has proceeded into human trials with some concerns like renal impairment. NOS immune interventions and modulation of neuroinflammation via microglial polarization further underscore the therapeutic potential of targeting NO/NOS. Indirect interventions, including mild hypothermia, medical gases (e.g., hydrogen sulfide, hyperbaric oxygen), zinc ions, and compounds like Ebselen, also show promise by regulating NO/NOS activity and mitigating secondary injury. Moreover, despite these advances, limitations persist, such as inconsistent study outcomes, narrow therapeutic windows, and the need for more clinical validation. Future directions should focus on optimizing intervention timing, exploring combinatorial therapies, and bridging preclinical findings with clinical applications to improve TBI outcomes.

## Prospects

As research into the roles of NO and NOS in TBI advances, it becomes increasingly imperative to delineate their complex involvement in core pathological processes, particularly neuroinflammation, oxidative stress, and apoptosis. A deeper, more mechanistically nuanced understanding of the NO/NOS axis not only enriches our theoretical framework of TBI pathophysiology but also holds the key to designing targeted, precision-based therapeutic strategies. Despite compelling preclinical evidence, clinical investigation of NO/NOS-modulating agents in TBI remains notably limited. This gap underscores an urgent need for rigorously designed, well-controlled clinical trials to evaluate the safety, dosing, and efficacy of such interventions in human patients. Given the inherently multidisciplinary nature of TBI research, spanning neuroscience, molecular biology, clinical medicine, and bioengineering, the development of next-generation NO-related therapeutics, including NOS inhibitors and NO-releasing devices, demands integrated expertise across these domains. Moreover, the interplay between NO and other medically relevant gases (e.g., hydrogen sulfide, carbon monoxide, and xenon) represents a promising yet underexplored frontier that warrants systematic investigation. In this context, fostering robust interdisciplinary collaboration is not only beneficial but essential. Only through concerted, cross-domain efforts can the field advance NO-based approaches in TBI from mechanistic insight to transformative clinical application, ultimately achieving new levels of therapeutic sophistication and patient benefit.

## Conclusions and Limitations

The review concludes that NO plays a dual role in TBI, offering neuroprotection at physiological levels but causing cytotoxicity and secondary injury when overproduced (**Figure 1**). While various NO-modulating therapies show promise in preclinical models, most lack clinical validation. The primary limitations of this review design include its reliance on narrative synthesis, which precludes quantitative pooling of data. Additionally, the inclusion criteria were restricted to English-language publications, potentially introducing language bias. The heterogeneity of animal models and outcome measures across the included preclinical studies also limits direct comparability. Finally, as most evidence is derived from rodent models, the translatability to human clinical trials remains uncertain.

**Figure 1:**
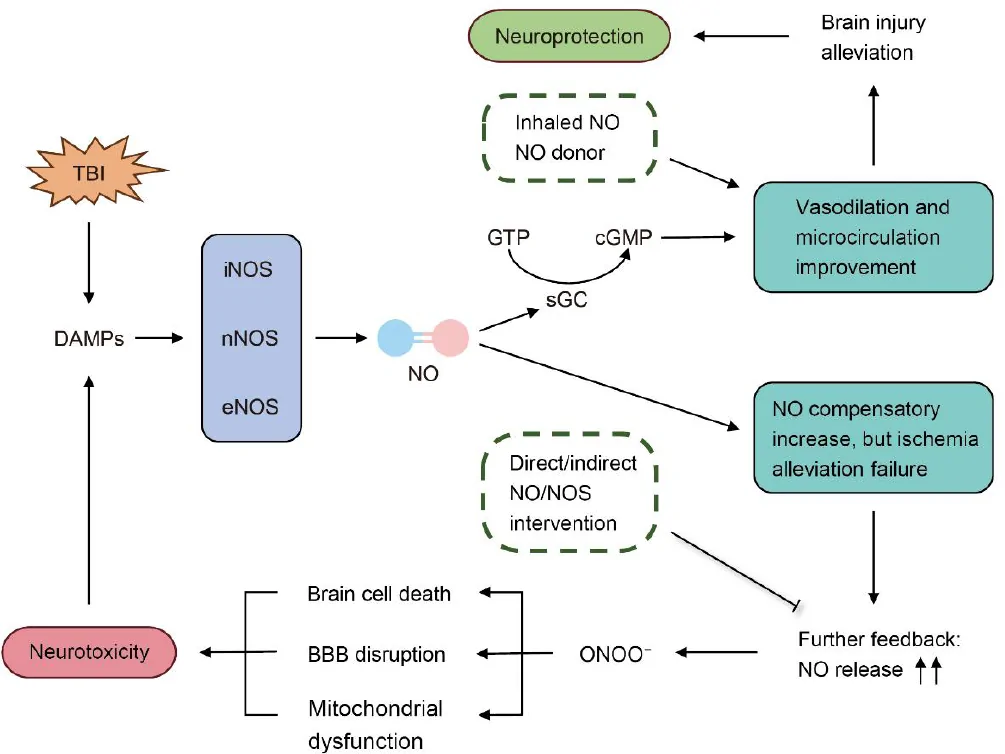
The “double-edged sword” NO signaling in TBI and potential intervention targets. This schematic illustrates the dual role of NO in TBI. It depicts how TBI triggers NO release via NOS isoforms, leading to either neuroprotection through vasodilation or neurotoxicity via peroxynitrite formation. The diagram further outlines potential therapeutic interventions targeting this pathway to alleviate brain injury. Created with Microsoft PowerPoint Office 2021. BBB: Blood–brain barrier; cGMP: cyclic guanosine monophosphate; DAMPs: damage-associated molecular patterns; eNOS: endothelial nitric oxide synthase; GTP: guanosine triphosphate; iNOS: inducible nitric oxide synthase; nNOS: neuronal nitric oxide synthase; NO: nitric oxide; NOS: nitric oxide synthase; ONOO-: peroxynitrite; sGC: soluble guanylyl cyclase; TBI: traumatic brain injury.

**Facts**

1. Nitric oxide (NO) in traumatic brain injury (TBI) is a “double-edged sword.”
2. Inhaled NO, NO donors, and nitric oxide synthase inhibitors (e.g., VAS203) improve cerebral blood flow and neurological outcomes, and reduce intracranial pressure.
3. TBI disrupts systemic NO homeostasis, affecting distant organs, such as the heart, via vagal activation and the formation of nitrosohemoglobin.

**Open Questions**

1. Why have preclinical NO interventions failed to translate into clinical practice? What are the barriers to human trials?
2. What are the precise therapeutic windows for targeting specific nitric oxide synthase isoforms, given their distinct roles in the phases of TBI?
3. How do microenvironments dictate the neuroprotective versus toxic effects of NO signaling? Can this be modulated?
4. Could NO modulators, when combined with other therapies (e.g., mild hypothermia, medical gases like hydrogen sulfide, or zinc ions), achieve synergistic effects and overcome the limitations of monotherapy?

## Data Availability

*Not applicable.*
